# Electric field simulations of transcranial direct current stimulation in children with perinatal stroke

**DOI:** 10.3389/fnhum.2023.1075741

**Published:** 2023-02-02

**Authors:** Helen L. Carlson, Adrianna Giuffre, Patrick Ciechanski, Adam Kirton

**Affiliations:** ^1^Calgary Pediatric Stroke Program, Alberta Children’s Hospital, Calgary, AB, Canada; ^2^Alberta Children’s Hospital Research Institute (ACHRI), Calgary, AB, Canada; ^3^Hotchkiss Brain Institute, University of Calgary, Calgary, AB, Canada; ^4^Department of Pediatrics, University of Calgary, Calgary, AB, Canada; ^5^Department of Clinical Neuroscience and Radiology, University of Calgary, Calgary, AB, Canada

**Keywords:** pediatric, MRI, hemiparesis, cerebral palsy, tDCS, neuromodulation, current, electric field

## Abstract

**Introduction:**

Perinatal stroke (PS) is a focal vascular brain injury and the leading cause of hemiparetic cerebral palsy. Motor impairments last a lifetime but treatments are limited. Transcranial direct-current stimulation (tDCS) may enhance motor learning in adults but tDCS effects on motor learning are less studied in children. Imaging-based simulations of tDCS-induced electric fields (EF) suggest differences in the developing brain compared to adults but have not been applied to common pediatric disease states. We created estimates of tDCS-induced EF strength using five tDCS montages targeting the motor system in children with PS [arterial ischemic stroke (AIS) or periventricular infarction (PVI)] and typically developing controls (TDC) aged 6–19 years to explore associates between simulation values and underlying anatomy.

**Methods:**

Simulations were performed using SimNIBS https://simnibs.github.io/simnibs/build/html/index.html using T1, T2, and diffusion-weighted images. After tissue segmentation and tetrahedral mesh generation, tDCS-induced EF was estimated based on the finite element model (FEM). Five 1mA tDCS montages targeting motor function in the paretic (non-dominant) hand were simulated. Estimates of peak EF strength, EF angle, field focality, and mean EF in motor cortex (M1) were extracted for each montage and compared between groups.

**Results:**

Simulations for eighty-three children were successfully completed (21 AIS, 30 PVI, 32 TDC). Conventional tDCS montages utilizing anodes over lesioned cortex had higher peak EF strength values for the AIS group compared to TDC. These montages showed lower mean EF strength within target M1 regions suggesting that peaks were not necessarily localized to motor network-related targets. EF angle was lower for TDC compared to PS groups for a subset of montages. Montages using anodes over lesioned cortex were more sensitive to variations in underlying anatomy (lesion and tissue volumes) than those using cathodes over non-lesioned cortex.

**Discussion:**

Individualized patient-centered tDCS EF simulations are prudent for clinical trial planning and may provide insight into the efficacy of tDCS interventions in children with PS.

## Introduction

Perinatal stroke is the leading cause of unilateral cerebral palsy (CP), characterized by motor disability affecting independence and quality of life (Kirton and deVeber, 2013). Stroke in the perinatal period typically results from an occlusion of a cerebral artery, commonly the middle cerebral artery (arterial ischemic stroke, AIS), or from a subcortical venous infarction damaging periventricular white matter (periventricular venous infarction, PVI) (Dunbar and Kirton, 2018). Perinatal stroke is relatively common (1:1100) (Dunbar et al., 2020) but despite this, there are no prevention strategies thus substantial disability and healthcare burden will likely continue. Because perinatal stroke occurs at or near birth (between 20 weeks gestation and 28 days of life) (Raju et al., 2007), and is often unilateral, this patient group affords a unique opportunity to investigate developmental and interventional neuroplasticity of the motor system in an otherwise healthy brain. Subsequent motor impairments last a lifetime and improving motor function as early as possible is crucial to leverage maximal neuroplasticity.

Complementary to traditional physical rehabilitation interventions for motor dysfunction, transcranial direct current stimulation (tDCS), a non-invasive brain stimulation (NIBS) technique, is emerging as a potentially effective therapy tool. tDCS induces a weak electric current on the scalp that extends to underlying neuronal tissue located between the anode and cathode. These currents alter cortical excitability in a polarity-dependent way (Nitsche and Paulus, 2000) and when paired with a behavioral task such as intensive motor therapy, may facilitate neuroplasticity that persists beyond the stimulation period (Stagg and Nitsche, 2011). Conventional tDCS montages typically use one anode and one cathode, whereas high-definition tDCS (HD-tDCS) montages use an array of electrodes to increase the focality of current flow (Datta et al., 2009; Dmochowski et al., 2011). Despite extensive study, the underlying mechanisms and high inter-subject variability of tDCS are poorly understood and may depend on experimental parameters including stimulation intensity, duration, repetition rate, electrode montage, configuration (To et al., 2016), as well as other inter-individual factors such as anatomy, neurochemistry, neurophysiology, and more (Li et al., 2015; Vergallito et al., 2022). Whether the stimulation was online (during the performance of a task) or offline (before/after performing a task) also may modulate tDCS effects (Stagg et al., 2011; Bikson et al., 2013). Age is an important additional factor throughout the lifespan (Moliadze et al., 2015) where studies have shown opposite tDCS polarity effects in both children and the elderly (Moliadze et al., 2018; Ghasemian-Shirvan et al., 2022).

Despite these sources of variability, multiple clinical trials have successfully demonstrated safety and benefits for a number of neurological and psychiatric conditions, including improving motor function in adults after stroke (Bastani and Jaberzadeh, 2012; Butler et al., 2013; Elsner et al., 2016; Kang et al., 2016). We have previously shown that both conventional tDCS, and HD-tDCS, targeting the primary motor cortex (M1) can enhance motor learning in typically developing children (Ciechanski and Kirton, 2017; Cole et al., 2018). Early evidence from recent controlled trials suggests possible efficacy in improving upper limb motor function in pediatric patients with unilateral CP (Kirton et al., 2016a; Inguaggiato et al., 2019; Salazar Fajardo et al., 2021). Given the promising results of such trials, individualized estimates of tDCS-induced electric fields, taking into account lesion location and extent, could shed light on differences between responders and non-responders, though are not commonly done.

In contrast to the relative ease of tDCS intervention methods, the calculations required to estimate current flow within the brain are challenging, particularly in the developing brains of children (Datta et al., 2013; Shahid et al., 2014; Opitz et al., 2015), but nonetheless may be informative. Idiosyncrasies of gyral/sulcal geometry, white (WM) and gray matter (GM) architecture, and variations in skull thickness make accurate head model reconstructions and tissue segmentations difficult but doable (Datta et al., 2009; Opitz et al., 2015). Such individualized modeling is imperative to maximize the therapeutic potential of tDCS. Nearly all modeling studies to date have been conducted in adults though several recent studies in children revealed that children may be exposed to higher peak electric fields compared to adults using the same current intensity and that this varies by tDCS montage (Bikson et al., 2012a,b; Minhas et al., 2012; Kessler et al., 2013; Gillick et al., 2014; Ciechanski et al., 2018).

Currently, there is little existing evidence quantifying effects of perinatal stroke lesions on tDCS currents (Gillick et al., 2014). CSF is particularly conductive and differences in CSF architecture dramatically change tDCS current flow (Datta et al., 2009, 2011; Bikson et al., 2012a). The presence of large CSF-filled compartments after middle cerebral artery (MCA) ischemic stroke or changes in ventricle morphology after PVI are likely important determinants of current propagation as demonstrated in adult stroke (Datta et al., 2011; Dmochowski et al., 2013). Additionally, damage to gray and white matter due to stroke likely plays an important role. With the steady increase of clinical trials applying tDCS as a means to treat children with CP (Aree-uea et al., 2014; Gillick et al., 2015; Grecco et al., 2015; Moura et al., 2016; Kirton et al., 2017), it seems prudent to systematically estimate tDCS-induced electric fields in this population. While neuroimaging and simulation software may not be optimized for this purpose, with appropriate attention to detail, we may still obtain useful information to inform the clinical trial planning for tDCS in disabled children (Datta et al., 2011; Halko et al., 2011).

Accordingly, we created patient-specific estimates of tDCS-induced electric fields (EF), employing multiple conventional tDCS and HD-tDCS montages targeting the motor system. This was done in children with CP caused by perinatal AIS or PVI as well as in a group of typically developing control children (TDC) without stroke. We hypothesized that peak EF strength would be higher, and EF angle in motor cortex would be more variable, in children with AIS and PVI compared to TDC due to neuroanatomical differences and larger volumes of highly conductive CSF in perilesional areas. We also explored associations between anatomical factors and EF simulation values.

## Materials and methods

### Participants

This was a retrospective, cross-sectional, population-based study involving three participant groups, two groups with perinatal stroke (AIS or PVI) and one group of controls with no history of stroke (Table 1).

**TABLE 1:** Demographic and anatomical characteristics of participant groups.

Characteristics by participant group	AIS (*N* = 21)	PVI (*N* = 30)	TDC (*N* = 32)
Age–mean (SD) [min-max] years	13.7 (3.9) [6.6–19.5]	11.8 (3.3) [6.6–19.8]	13.4 (3.5) [6.5–19.0]
**Sex–N [%]**			
Male	*N* = 14 [66.7%]	*N* = 20 [66.7%]	*N* = 17 [53.1%]
Female	*N* = 7 [33.3%]	*N* = 10 [33.3%]	*N* = 15 [46.9%]
**Stroke hemisphere–N [%]**			
Left	*N* = 15 [71.4%]	*N* = 17 [56.7%]	**–**
Right	*N* = 6 [28.6%]	*N* = 13 [43.3%]	**–**
**Volumes–mean (SD) [min-max]**			
GM	0.48 (0.08) [0.20–0.57]	0.52 (0.03) [0.48–0.57]	0.51 (0.03) [0.46–0.55]
WM	0.30 (0.04) [0.18–0.36]	0.31 (0.02) [0.27–0.35]	0.32 (0.02) [0.29–0.36]
TIV (cm^3^)	1362 (140) [1122–1664]	1510 (145) [1283–1925]	1576 (169) [1236–1991]
Lesion (cm^3^)	37.2 (40.7) [1.1–161.0]	–	–
**Thickness–mean (SD) [min-max] mm**			
Skull thickness	9.8 (1.2) [7.2–12.0]	8.9 (1.6) [6.2–12.9]	9.4 (2.4) [5.78–18.1]
Scalp to GM surface	14.6 (4.3) [8.65–28.3]	11.4 (2.2) [8.3–16.6]	11.9 (2.5) [8.15–19.9]

SD, standard deviation; AIS, arterial ischemic stroke; PVI, periventricular venous infarction; TDC, typically developing controls; GM, gray matter; WM, white matter; TIV, estimated total intracranial volume (cm^3^). GM and WM volumes are proportions in relation to TIV. Thickness values reported here were measured over M1 in the lesioned hemisphere (right hemisphere in TDC).

Participants with perinatal stroke were recruited *via* the Alberta Pediatric Stroke Program, a population-based research cohort (Cole et al., 2017). Inclusion criteria were: (1) an MRI-confirmed diagnosis of unilateral PVI, neonatal arterial ischemic stroke (NAIS) or arterial presumed perinatal ischemic stroke (APPIS) reviewed by a pediatric neurologist using established criteria (Kirton et al., 2008); (2) aged between 6 and 19 years with term birth (>36 weeks); (3) hemiparesis as determined by a Pediatric Stroke Outcome Measure (PSOM) score >0.5 (Kitchen et al., 2012); (4) no history of other neurological conditions (besides stroke), diffuse or bilateral injuries, and (5) no MRI contraindications. Subsequently, stroke patients were grouped according to their mechanism of injury as either PVI or AIS (combined NAIS and APPIS groups).

TDC volunteers with no history of perinatal stroke were recruited through a community-based healthy controls recruitment program.^2^ All TDC participants were right-handed as per the Edinburgh Handedness Inventory (Oldfield, 1971), aged between 6 and 19 years, and had no neurological conditions or MRI contraindications. Sex and age (±1 year) of TDC were comparable to AIS and PVI participants.

Prior to participation, both written informed parental consent and participant assent were obtained in accordance with the University of Calgary Research Ethics Board that approved this study.

### Imaging

Magnetic resonance (MR) images were acquired at the Alberta Children’s Hospital Diagnostic Imaging Suite with a 3.0 Tesla General Electric MR750w MRI scanner (GE Healthcare, Waukesha, WI, USA) and a 32-channel head coil. High-resolution anatomical T1-weighted fast spoiled gradient echo (FSPGR) images were acquired in the axial plane [minimum of 166 slices, no skip; voxel size = 1.0 mm isotropic; repetition time (TR) = 8.5 ms; echo time (TE) = 3.2 ms; flip angle = 11*^o^*; matrix = 256 × 256]. T2-weighted images were acquired in the axial plane [36 slices, no skip; voxel size = 0.45 × 0.45 mm; slice thickness = 3.6 mm; TR/TE = 6187/80 ms; matrix = 512 × 512]. Diffusion-weighted images (DWI) were acquired in 32 non-collinear directions (*b* = 750 s/mm^2^, 3 volumes using *b* = 0 s/mm^2^, voxels = 2.2 mm isotropic, duration = 6 min, TR/TE = 11.5 s/70 ms).

### Preprocessing and volume conductor modeling

**FIGURE 1:**
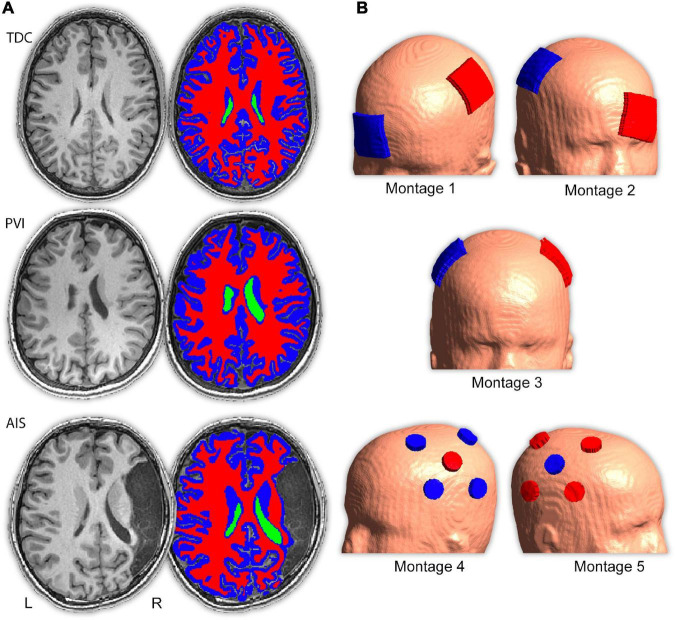
Image processing procedure and montage electrode placements. **(A)** T1- and T2-weighted images were segmented into five tissue types (only T1-weighted images, ventricle, gray and white matter masks are shown here) and converted to a tetrahedral mesh *via* the headreco command. Three example participants are shown; a typically developing control (TDC), a child with periventricular venous infarction (PVI) and a child with arterial ischemic stroke (AIS). Areas of cerebrospinal fluid (CSF)-filled lesion and dilated ventricles were coded as CSF. **(B)** Five transcranial direct-current stimulation (tDCS) montages targeted at enhancing motor function were simulated. Electrode positions are illustrated using a child with a left hemisphere stroke. Red electrodes denote anode locations, blue electrodes denote cathode locations.

Preprocessing and modeling was performed using SimNIBS ver 3.2.3 (Thielscher et al., 2015)^3^ using a standard pipeline. The Computational Anatomy Toolbox (CAT12)^4^ and Statistical Parametric Mapping (SPM12)^5^ were used to segment T1- and T2-weighted anatomical volumes into five tissue types, correct segmentation errors, and generate the meshed model *via* the SimNIBS *headreco* command (Windhoff et al., 2013; Thielscher et al., 2015). Segmented tissue types were gray (GM) and white matter (WM), cerebrospinal fluid (CSF), skull, and scalp (Figure 1). While CAT12, SPM12, and *headreco* are not optimized for use with non-normal brain anatomies (such as those with lesions) or for children, our experience was that the majority of the anatomical images were accurately segmented. Additional care was taken to ensure that tissue segmentations were accurate *via* slice-by-slice examination of all five segmentation masks overlaid on T1-weighted anatomy using ITK-SNAP for visualization (Yushkevich et al., 2006). Some children with AIS had lesioned areas filled with CSF that were misclassified as GM or WM, likely due to use of tissue probability priors, and these participants were necessarily excluded (details in results). For PVI patients, we ensured that dilated ventricles were correctly classified as CSF and participants were again excluded as necessary. *Via* the *dwi2cond* command, diffusion scans were preprocessed (eddy current and head motion corrected), co-registered to the T1-weighted anatomical image, and diffusion tensors were calculated.

Subsequently, tetrahedral volume mesh head models were created using SimNIBS (Windhoff et al., 2013) and visualized *via* Gmsh (Geuzaine and Remacle, 2009; Figure 1). tDCS-induced EF estimations were based on the finite element model (FEM). Previously established conductivity values for each tissue type were utilized (Wagner et al., 2004; Opitz et al., 2015): GM (0.275 S/m), CSF (1.654 S/m), bone (0.010 S/m), and scalp (0.465 S/m). While these values are not specifically optimized for children, since conductivity values vary with age due to changes in tissue water content, it has been demonstrated that conductivity values for white and gray matter by the age of 6 years approximate that of adults (Mohammed et al., 2017). For WM, diffusion tensors provided personalized estimates of anisotropic conductivities, previously demonstrated to have a significant effect on electric field calculations (Ciechanski et al., 2018). These anisotropic conductivity values were calculated using the direct mapping method by which the direction and size of tensors are based on underlying diffusion characteristics (Opitz et al., 2011). A linear relationship has been previously established between the diffusion tensor eigenvalues and conductivity tensors that can be used to rigorously infer conductivity values (Tuch et al., 2001). While this linear relationship has not specifically been investigated in children, diffusion tensor eigenvalues, diffusion anisotropy, and resulting tensor metrics have been shown to vary in predictable ways during maturation of white matter (Beaulieu, 2002; Lebel and Beaulieu, 2011) suggesting that conductivity values calculated on the basis of diffusion tensor eigenvalues are reflective of underlying white matter tissue conductivity.

### Electrode placements

Electric fields distributions for five tDCS montages commonly used in stroke motor rehabilitation aimed at enhancing function of a paretic upper extremity were simulated (Figure 1 and Supplementary Table 1). Since strokes can occur in either hemisphere, montages were customized using stroke laterality for each participant. Lesioned M1 is referred to as M1_*Les*_, and non-lesioned M1 as M1_*NonLes*_. For our right-handed control participants, the left hemisphere was used as M1_*NonLes*_, and the right as M1_*Les*_, thus the montage configuration is the same as for participants with a stroke in the right hemisphere, simulating interventions intended to improve function in the left (non-dominant) hand.

Montage 1 simulated anodal tDCS of M1_*Les*_. The anode was centered over the “hand-knob” area (Yousry et al., 1997) of M1_*Les*_ corresponding to the C3 or C4 electrode in the standardized EEG 10/20 system (depending on the side of stroke for each individual). The cathode was positioned over the contralateral supraorbital area (Fp1 or Fp2). Montage 2 simulated cathodal tDCS of M1_*NonLes*_. The cathode was centered over the hand-knob area of M1_*NonLes*_ (C3 or C4), and the anode over the contralateral supraorbital area (Fp1 or Fp2). Montage 3 simulated anodal tDCS of M1_*Les*_ *via* application of bihemispheric tDCS of both M1 areas. The anode was centered over the hand-knob area (C3 or C4) of M1_*Les*_, and the cathode over the hand-knob area in the opposite hemisphere (M1_*NonLes*_ C3 or C4). Montage 4 simulated anodal tDCS of M1_*Les*_ using high-definition (HD) tDCS. The anode was centered on M1_*Les*_ and four cathodes were equally spaced surrounding the anode depending on the stroke laterality. For children with a stroke in the left hemisphere, the anode was placed on C3 and cathodes on CP5, FC5, FC1, and CP1. For children with a stroke in the right hemisphere (and controls), the anode was placed on C4 and cathodes on CP6, FC6, FC2, and CP2. Montage 5 simulated cathodal tDCS of M1_*NonLes*_ using HD-tDCS. This montage had similar electrode placements as montage 4 but the anodes and cathodes were placed over M1_*NonLes*_ and were reversed such that the center electrode was the cathode and the surrounding electrodes were anodes.

### Electrode configuration

For montages 1–3, simulations used a 5 × 5 cm rubber electrode of 1 mm thickness enclosed in a saline-soaked, 6 mm thick, 5 × 5 cm sponge with a connector area of 2.5 × 0.5 cm, centered along the posterior aspect. Rubber electrode conductivity was set to 29.4 S/m, and saline-soaked sponges to 1.0 S/m. Current was set to + 1 mA for the anode and −1 mA for the cathode.

For HD-tDCS montages 4 and 5, electrodes were modeled to simulate placement using a cap with pre-existing electrode spaces (Villamar et al., 2013; Giuffre et al., 2019). Electrodes were circular (external diameter = 2.4 cm), 6.5 mm thick, with a gel thickness of 1.0 mm. Gel conductivity was set to 1.0 S/m and remaining conductivity values were used as above. For montage 4, anode current was + 1.0 mA and cathodes were each −0.25 mA. For montage 5, cathode current was −1.0 mA and surrounding anodes were each + 0.25 mA.

### Electrical field simulation values

Variables of interest were extracted for each participant for each tDCS montage. Peak EF strength (in V/m) at any point in space within the GM and WM tissue masks was extracted for each of the five tDCS montages. These values corresponded to the 99.9th percentile value in each parametric map. Field focality was extracted as the volume of GM or WM tissue (in cm^3^) that had EF strength values ≥75% of the 99.9th percentile whereby higher values reflect lower field focality. In addition, two spherical regions of interest (ROI), 20 mm in diameter, were used to extract mean EF strength measurements in bilateral primary motor cortices [LM1: −38 −26 62; RM1: 38 −22 62] for GM and WM tissue. ROIs were positioned in M1 to explore EF simulation values within motor cortices since the tDCS montages were aimed at modulating motor-cortex excitability. The volumes of GM and WM tissue within these ROIs were additionally extracted to use as covariates.

Electric fields angles were extracted from target M1 for each montage using customized versions of open-source Matlab scripts from a previous study (Evans et al., 2022). For montages 1, 3, and 4, M1_*Les*_ was the target ROI, for montages 3 and 5 M1_*NonLes*_ was the target. EF angles represent the angle (expressed in radians) between the cortical surface normal vector and the EF vector in the middle gray matter surface for each vertex. Whole-brain parametric surface maps containing EF angles were generated by SimNIBS and were sampled using a surface-based region of interest placed on the posterior bank of the precentral gyrus “hand knob” area (Yousry et al., 1997) in the target hemisphere. The central target point was identified in gray matter using each participant’s high-resolution T1-weighted image and the nearest vertex on the angle surface map was located using the k-nearest neighbor function (knnsearch) in Matlab. The surrounding vertices were then selected by dilating the ROI from its center point to encompass the nearest 5 vertices in all directions along the surface. Angle values from each vertex within the ROI were extracted and averaged together to represent EF angle.

### Anatomical characteristics

Participants’ anatomical characteristics were extracted to explore associations with simulation values and for use as statistical covariates. Relative whole-brain tissue volumes for each participant were calculated using binary masks from tissue segmentation. Total intracranial volume (TIV) was calculated as the sum of the volumes of the WM, GM, and CSF masks (in cm^3^). Relative GM and WM volumes (in cm^3^) were calculated as GM_*vol*_ = GM_*raw*_/TIV and WM_*vol*_ = WM_*raw*_/TIV, respectively. For children with AIS, lesion volumes were measured from the native T1 image using an intensity-based semi-automated 3D fill tool in MRIcron (Rorden et al., 2007) and are expressed in cm^3^. Skull thickness and distance between the scalp surface and the GM surface (in mm) were measured in both hemispheres using the measuring tool in ITK-Snap viewing a coronal slice of the T1 image with the GM tissue mask overlaid. Distances were measured at participant-specific electrode coordinates centered over M1_*Les*_ and M1_*NonLes*_ using a plane perpendicular to the tangent of the skull surface.

### Statistical analyses

Statistics were performed using Jamovi version 1.6.23 (Jamovi., 2021) and SPSS (IBM, version 28, USA). Distribution normality was assessed using Shapiro-Wilk and subsequent analyses were parametric or non-parametric as appropriate. Differences in demographic variables between groups were examined using one-way analysis of variance (ANOVA) for age and Chi-square for sex and stroke hemisphere. Group differences in tissue volumes (GM, WM), TIV and thickness values were assessed using Kruskal-Wallis or ANOVA followed by pairwise comparisons. Associations between age and tissue volumes were explored with Spearman’s rho for each participant group separately.

Quade’s non-parametric analyses of covariance (ANCOVAs), followed by pairwise *t*-tests, explored group differences in simulation values (peak EF strength, field focality) including age as a covariate. Quade’s ANCOVAs were also used to examine group differences in mean EF within the target M1 ROI for each montage using tissue volume within the ROI as a covariate. For montages 1, 3 and 4, M1_*Les*_ was the target ROI, for montages 3 and 5 M1_*NonLes*_ was the target. Friedman’s ANOVAs (followed by pairwise comparisons) were performed to assess within-group differences in simulation values between the five montages. For EF angles, Kruskal–Wallis tests explored group differences for each montage followed by Dwass-Steel-Critchlow-Flinger (DSCF) pairwise contrasts. Levene’s test for homogeneity of variance explored differences in distribution variability for each patient group and montage.

To explore associations between anatomical characteristics (tissue volumes, lesion volumes, skull thickness, and scalp to GM surface distance) and simulation values (peak EF strength, field focality, mean EF strength in M1 ROIs, EF angle), partial Spearman’s rho analyses (controlling for age) were performed for each montage and for each participant group separately. For mean EF strength in the M1 ROIs, age and GM and WM volumes within the M1 ROIs were used as factors rather than whole brain tissue volumes.

For all analyses, multiple comparisons were corrected using False Discovery Rate (FDR) corrections using *q* = 0.05 (Benjamini and Hochberg, 1995).

## Results

### Population

**FIGURE 2:**
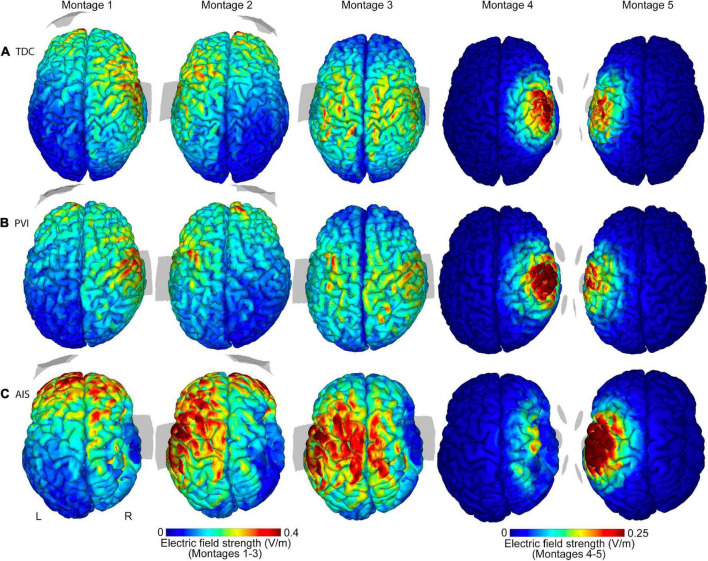
Illustrations of electric field (EF) strength distributions on the gray matter surface for five transcranial direct-current stimulation (tDCS) montages. Shown are three individual participants of similar ages (15.6 y, 14.4 y, and 15.0 y, respectively) from the **(A)** TDC, **(B)** PVI, and **(C)** AIS groups. T1-weighted anatomy is also illustrated in Figure 1 for the same participants. Stroke participants have a right hemisphere stroke. Note that the EF strength scales are the same for each participant group but are different between montages 1–3 and montages 4–5. Gray shaded areas represent electrode placements. TDC, typically developing control; PVI, periventricular infarction; AIS, arterial ischemic stroke.

A total of 107 participants were recruited, however, 24 participants (22%) were excluded (19 AIS and 5 PVI) due to excessive head motion (*N* = 2 AIS), failure of the modeling pipeline (*N* = 3 AIS), or misclassification of brain tissue (total *N* = 19; comprising PVI *N* = 5, AIS *N* = 14). Excluded participants were generally younger (<10 years) and had larger lesions than included participants. Current flow simulations were successfully performed in a final sample of 83 participants (Table 1 and Figure 2), including 32 TDC, 21 AIS and 30 PVI.

Participant demographics did not differ between groups [age: *F*_(2_,_80)_ = 2.2, *p* = 0.12; sex: χ^2^_(2,N=83)_ = 1.5, *p* = 0.47; stroke hemisphere: χ^2^_(1,N=51)_ = 1.2, *p* = 0.28]. Tissue volumes were significantly different between groups [GM: *H*_(2)_ = 8.3, *p* = 0.016; WM: *H*_(2)_ = 15.4, *p* < 0.001; TIV: *F*_(2_,_80)_ = 12.5, *p* < 0.001] such that GM volumes were highest in the TDC and PVI groups and smallest in the AIS group compared to PVI (*p* = 0.03) with no other group differences noted. WM volumes were highest in the TDC group compared to both AIS (*p* = 0.001) and PVI (*p* = 0.009) but the AIS and PVI groups did not differ from each other (*p* = 0.52). TIV was smallest in the AIS group compared to both TDC (*p* < 0.001) and PVI (*p* = 0.003). TIV was not different between TDC and PVI (*p* = 0.21). Skull thickness was not different in either hemisphere between groups. In the lesioned hemisphere, scalp to GM surface distance was higher in the AIS group compared to both TDC (*p* = 0.013) and PVI (*p* = 0.004).

### Age and volume correlations

Age was negatively associated with GM volume for all patient groups (AIS: r_*s*_ = −0.63, *p* = 0.003; PVI: r_*s*_ = −0.61, *p* < 0.001; TDC: *r*_*s*_ = −0.70, *p* < 0.001) and was positively associated with WM volume for the TDC (*r*_*s*_ = 0.69, *p* < 0.001) and PVI (*r*_*s*_ = 0.48, *p* = 0.008) groups but not for AIS (*r*_*s*_ = −0.12, *p* = 0.62). TIV was not associated with age for any group (AIS: *r* = −0.25, *p* = 0.28; PVI: *r* = 0.30, *p* = 0.10; TDC: *r* = −0.11, *p* = 0.55). Age was subsequently used as a covariate in group analyses.

### Group differences in simulation values

**FIGURE 3:**
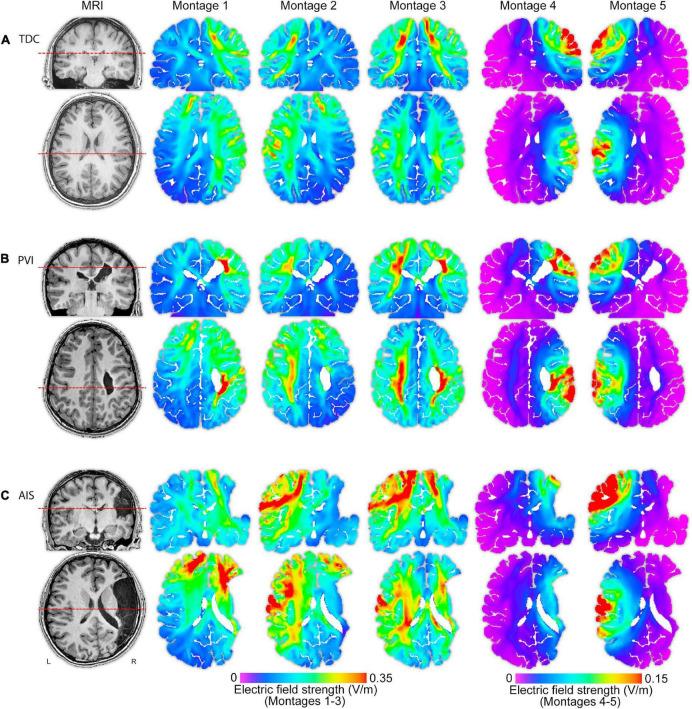
T1-weighted anatomical images and electric field (EF) strength (V/m) parametric maps for five montages for three individual participants (of similar ages 13.5, 13.1, and 15.0 years) from each of the **(A)** TDC, **(B)** PVI, and **(C)** AIS groups. These images illustrate that EF peak strengths, peak locations and field distributions vary idiosyncratically across patients and montages. The same MRI slices (denoted by dotted line) for each participant are shown in each row, though slice position varies between participants based on individual anatomy. Note that the EF strength scales are the same within montages 1–3 though different from montages 4–5. TDC, typically developing control; PVI, periventricular infarction; AIS, arterial ischemic stroke.

**FIGURE 4:**
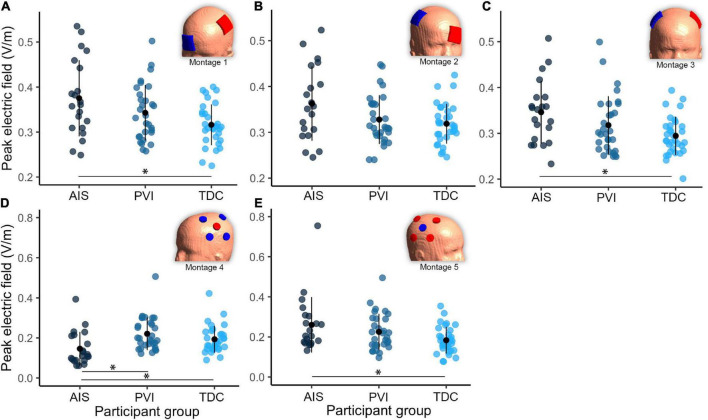
Group differences in peak electric field (EF) within gray matter between three participant groups and five transcranial direct-current stimulation (tDCS) montages **(A–E)**. Note that the EF strength scales are different between montages 1–3 and montages 4–5. Montage diagrams illustrate electrode configuration for a child with a left hemisphere stroke. AIS, arterial ischemic stroke; PVI, periventricular venous infarction; TDC, typically developing control. Horizontal lines denote significant pairwise contrasts (*p_FDR_ < 0.01), vertical lines denote ± 1 standard deviation. Red electrodes denote anode locations, blue electrodes denote cathode locations.

Differences in simulation values were found among the three participant groups (Table 2 and Figures 2–4). For montages 1, 3 and 5, peak EF strength was higher in the AIS group compared to TDC [montage 1: *t*_(80)_ = 2.6, *p* = 0.011; montage 3: *t*_(80)_ = 2.9, *p* = 0.005; montage 5: *t*_(80)_ = 2.6, *p* = 0.010]. Montage 4 showed higher peak EF strength in both the PVI [*t*_(80)_ = 4.0, *p* < 0.001] and TDC [*t*_(80)_ = 2.8, *p* = 0.006] groups compared to AIS.

**TABLE 2:** Gray matter peak electric field strength and field focality for five tDCS montages by participant group.

Values–mean (SD) [min-max]	AIS (*N* = 21)	PVI (*N* = 30)	TDC (*N* = 32)
**Peak electric field strength (in V/m)**			
Montage 1–anodal tDCS of M1_Les_	0.38 (0.09) [0.25–0.54]	0.34 (0.06) [0.26–0.50]	0.32 (0.05) [0.23–0.40]
Montage 2–cathodal tDCS of M1_NonLes_	0.37 (0.10) [0.25–0.60]	0.33 (0.05) [0.24–0.45]	0.32 (0.04) [0.25–0.43]
Montage 3–bihemispheric tDCS	0.35 (0.07) [0.23–0.51]	0.32 (0.06) [0.25–0.50]	0.29 (0.04) [0.20–0.39]
Montage 4–anodal HD-tDCS of M1_Les_	0.15 (0.08) [0.06–0.39]	0.22 (0.08) [0.12–0.51]	0.19 (0.07) [0.09–0.42]
Montage 5–cathodal HD-tDCS of M1_NonLes_	0.26 (0.14) [0.13–0.76]	0.23 (0.09) [0.10–0.50]	0.18 (0.07) [0.08–0.35]
**Focality of electric field (cm^3^)**			
Montage 1–anodal tDCS of M1_Les_	9.2 (4.2) [0.9–16.8]	11.3 (4.4) [1.8–19.0]	13.3 (5.2) [2.9–29.7]
Montage 2–cathodal tDCS of M1_NonLes_	10.7 (5.5) [1.1–21.4]	13.9 (3.9) [7.0–21.5]	12.2 (4.3) [4.7 × 10^–5^–21.0]
Montage 3–bihemispheric tDCS	11.5 (5.2) [2.2–22.4]	15.1 (4.3) [6.9–23.5]	16.2 (5.1) [4.6–25.7]
Montage 4–anodal HD-tDCS of M1_Les_	3.9 (1.7) [0.8–6.8]	4.6 (1.2) [3.0–8.1]	5.5 (1.2) [2.6–7.9]
Montage 5–cathodal HD-tDCS of M1_NonLes_	4.2 (1.3) [2.0–6.7]	5.0 (1.4) [2.4–9.2]	5.6 (1.6) [2.2–9.0]

tDCS, transcranial direct current stimulation; AIS, arterial ischemic stroke; PVI, periventricular venous infarction; TDC, typically developing controls; GM, gray matter; WM, white matter; HD, high-definition tDCS. Peak electric field strength (EF) is reported in V/m. Peak values correspond to the 99.9th percentile value. Field focality is the tissue volume (in cm^3^) that had EF values ≥75% of the 99.9th percentile. Corresponding data for WM can be found in Supplementary Table 2.

For EF focality in montage 1, the volume of GM tissue above 75% of the 99.9th percentile was lower for the AIS group [*t*_(80)_ = 3.1, *p* = 0.002] compared to TDC. For montages 3–5, the AIS group showed lowest volumes compared to the TDC group [AIS vs. TDC: montage 3: *t*_(80)_ = 3.5, *p* = 0.001, montage 4: *t*_(80)_ = 4.0, *p* < 0.001, montage 5: *t*_(80)_ = 3.3, *p* = 0.002] and PVI group for montage 3 [AIS vs. PVI: *t*_(80)_ = 2.3, *p* = 0.022].

### Group differences in mean electric field strength within M1 regions of interest

**FIGURE 5:**
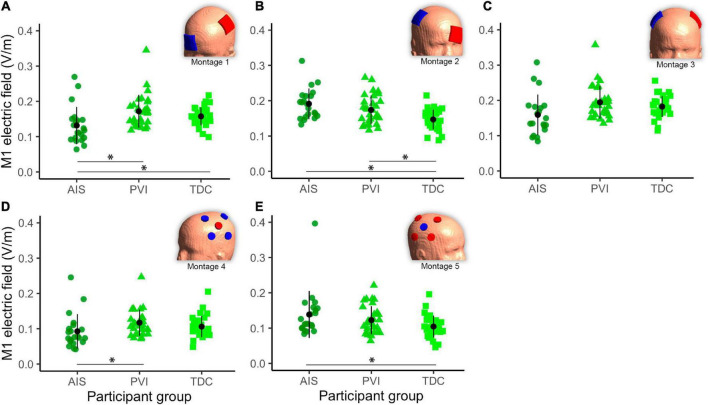
Mean electric field (EF) in gray matter (GM) of the target primary motor cortex (M1) ROI for five montages by participant group **(A–E)**. For montages 1, 3, and 4 lesioned M1 was the target, for montages 3 and 5 non-lesioned M1 was the target. Montage diagrams illustrate electrode configuration for a child with a left hemisphere stroke. ROI–region of interest, AIS–arterial ischemic stroke, PVI–periventricular venous infarction, TDC–typically developing control. Horizontal lines denote significant pairwise contrasts (*p_FDR_ < 0.01), vertical lines denote ± 1 standard deviation. Red electrodes denote anode locations, blue electrodes denote cathode locations.

Different mean EF strengths in GM within target M1 ROIs were found across participant groups (Figure 5). For montage 1, AIS showed lower mean EF strength compared to both TDC [*t*_(80)_ = 2.6, *p* = 0.01] and PVI [*t*_(80)_ = 3.0, *p* = 0.003] groups. For montage 2, TDC showed lower mean EF values compared to both AIS [*t*_(80)_ = 4.0, *p* < 0.001] and PVI [*t*_(80)_ = 2.8, *p* = 0.006]. For montage 4 AIS showed lower mean EF strength than for PVI [*t*_(80)_ = 2.6, *p* = 0.01] and for montage 5 AIS showed higher mean EF strength than TDC [*t*_(80)_ = 2.5, *p* = 0.02].

### Montage differences in electric field strength

**FIGURE 6:**
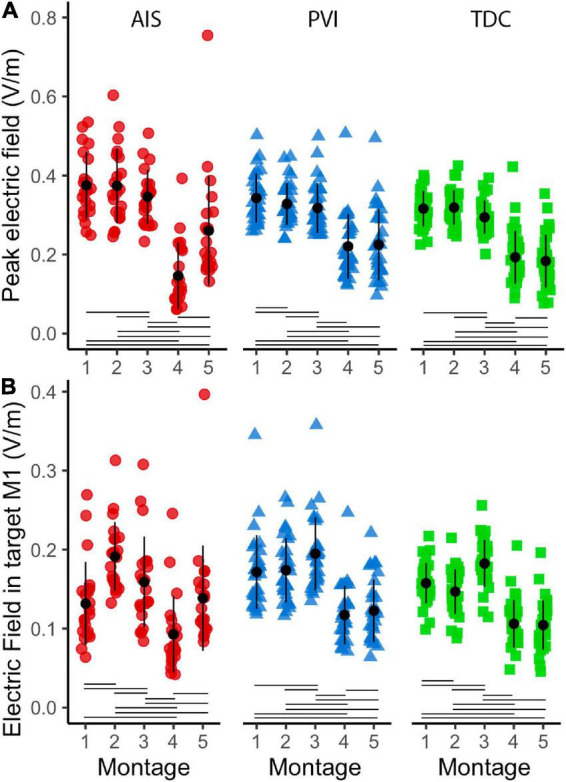
Peak electric field (EF) strength and mean EF in target ROI gray matter (GM) in five montages by participant group **(A)**. Peak EF in GM differed across montages as did mean electric field in target M1 ROIs **(B)**. For montages 1, 3, and 4, lesioned M1 was the target, for montages 3 and 5 non-lesioned M1 was the target. ROI, region of interest; AIS, arterial ischemic stroke (red circles); PVI, periventricular venous infarction (blue triangles); TDC, typically developing control (green squares); M1, primary motor cortex. Horizontal lines denote significant pairwise contrasts (*p*_FDR_ < 0.01), vertical lines denote ± 1 standard deviation.

Both peak EF strength and mean EF strength in target M1 ROIs differed widely among montages (Figure 6). The two HD montages (4 and 5) showed lowest peak EF strength compared to the other montages (1–3).

### Group differences in EF angle within M1 regions

**FIGURE 7:**
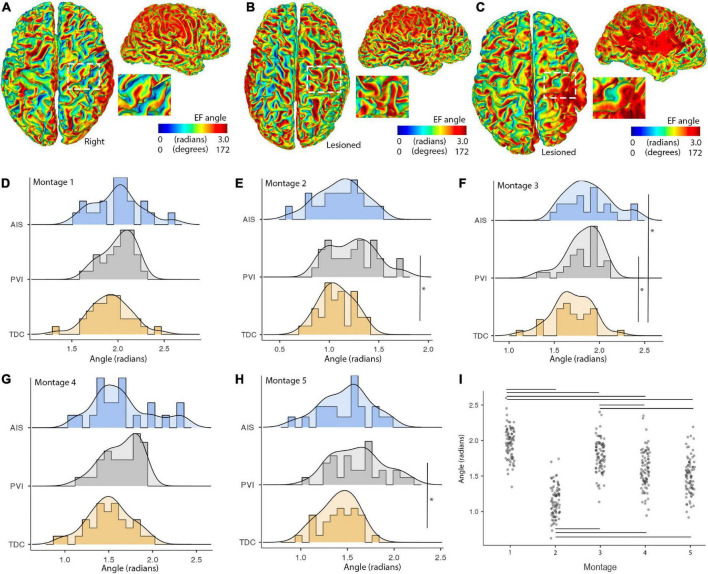
Illustrations of montage- and group-specific differences in electric field (EF) angles. Individual surface maps of EF angle for montage 1 are shown for panel **(A)**. A TDC participant **(B)**. A PVI participant and **(C)**. An AIS participant with insets displaying pre- and post-central gyrus of M1 in the lesioned/right hemisphere for each participant. **(D–H)** Show distributions and group differences in EF angles across the five montages. Vertical lines show significant differences between participant groups (*p < 0.05) **(I)**. EF angle varied widely across montage with montage 1 showing the highest and montage 2 showing the lowest EF angle values compared to all other montages. Horizontal lines show significant differences between montages (all *p*-values < 0.001). AIS, arterial ischemic stroke; PVI, periventricular venous infarction; TDC, typically developing control.

Electric fields angles within M1 posterior bank regions were different between participant groups for a subset of montages (Figure 7). Cathodal montages 2 and 5 showed lower EF angles for the TDC group compared to the PVI group (*W* = 3.78, *p* = 0.02). Montage 3 (bihemispheric) also showed lower EF angles for the TDC group compared to both the AIS (*W* = 3.5, *p* = 0.036) and PVI groups (*W* = 3.77, *p* = 0.021). Variability in EF angles were not different between patient groups for any montages. EF angles also varied over montage such that montage 1 showed higher angles and montage 2 showed lower angles compared to all other montages (all p-values < 0.001).

### Anatomical characteristics

Peak EF strength was not associated with relative whole-brain GM or WM tissue volume, scalp to GM distance or skull thickness for any participant group in any montage. Additionally Peak EF strength was not associated with lesion volume for AIS participants, within any montage. Focality volumes for EF were highly associated with GM and WM tissue volume and lesion volume for the AIS group (Table 3), specifically for montages 1, 3, and 4 that simulated using active anodes over M1_*Les*_. For the PVI group, GM volume was associated with EF focality (*r*_*s*_ = 0.63, *p* < 0.001) for montage 1 only. Mean EF in target M1 ROIs was associated with GM volume within those ROIs as well as lesion volumes for the AIS group, specifically for montages 1 and 3. Corresponding results from the white matter analyses are presented in Supplementary material. EF angles were not associated with anatomical characteristics.

**TABLE 3:** Associations between anatomical characteristics and simulation values for the AIS group (*N* = 21).

Montage factors	EF focality	EF in target M1
**Montage 1–anodal tDCS of M1_Les_**		
GM volume	*r*_s_ = 0.52, *p* = 0.02*	*r*_s_ = 0.51, *p* = 0.02*
WM volume	*r*_s_ = 0.32, *p* = 0.16	*r*_s_ = 0.13, *p* = 0.60
Lesion volume	*r*_s_ = −0.59, *p* = 0.006**	*r*_s_ = −0.53, *p* = 0.02*
**Montage 2–cathodal tDCS of M1_NonLes_**		
GM volume	*r*_s_ = 0.05, *p* = 0.82	*r*_s_ = 0.41, *p* = 0.08
WM volume	*r*_s_ = 0.25, *p* = 0.29	*r*_s_ = −0.12, *p* = 0.63
Lesion volume	*r*_s_ = −0.20, *p* = 0.40	*r*_s_ = −0.06, *p* = 0.81
**Montage 3–bihemispheric tDCS**		
GM volume	*r*_s_ = 0.66, *p* = 0.001**	*r*_s_ = 0.63, *p* = 0.003**
WM volume	*r*_s_ = 0.35, *p* = 0.13	*r*_s_ = 0.16, *p* = 0.50
Lesion volume	*r*_s_ = −0.45, *p* = 0.05	*r*_s_ = −0.58, *p* = 0.007**
**Montage 4–anodal HD-tDCS of M1_Les_**		
GM volume	*r*_s_ = 0.24, *p* = 0.31	*r*_s_ = 0.46, *p* = 0.04
WM volume	*r*_s_ = 0.60, *p* = 0.006**	*r*_s_ = 0.07, *p* = 0.77
Lesion volume	*r*_s_ = −0.63, *p* = 0.003**	*r*_s_ = −0.48, *p* = 0.03
**Montage 5–cathodal HD-tDCS of M1_NonLes_**		
GM volume	*r*_s_ = 0.43, *p* = 0.06	*r*_s_ = 0.25, *p* = 0.30
WM volume	*r*_s_ = 0.19, *p* = 0.43	*r*_s_ = −0.10, *p* = 0.68
Lesion volume	*r*_s_ = −0.10, *p* = 0.68	*r*_s_ = −0.07, *p* = 0.77

AIS, arterial ischemic stroke; GM, gray matter; WM, white matter; EF, electric field strength (in V/m). For EF focality, GM and WM volumes are whole brain proportions in relation to TIV. For EF in target M1, GM and WM are volumes within the M1 ROI only. r_s_–partial Spearman’s rho (controlling for age), *p_FDR_ < 0.05, **p_FDR_ < 0.001. Certain associations between anatomical and field characteristics were strong but were not statistically significant after multiple comparison correction.

## Discussion

We created patient-specific estimates of tDCS-induced EFs in a large sample of children with perinatal AIS or PVI, and controls, using individualized MRI and explored associations between anatomical characteristics and simulation values. Three tDCS and two HD-tDCS montages commonly used to enhance motor function were simulated. We demonstrate that montages utilizing active anodes over lesioned cortex had higher peak EF strengths for the AIS group compared to controls. In contrast, these montages showed lower mean EF strengths within target M1 regions suggesting that EFs were not consistently localized to motor network-related target brain areas. We also found that montages using active anodes over lesioned cortex were more sensitive to variations in underlying anatomy (lesion and tissue volumes) than those using cathodes over non-lesioned cortex. Individualized tDCS-induced EF simulations may provide insight into the efficacy of tDCS interventions in children with varying anatomy due to perinatal stroke and may inform future planning of interventions.

### Electrical field simulation values

Using simulations to identify areas of peak EF in a whole-brain manner may allow for quantification of tDCS-induced current “hotspots” (Minjoli et al., 2017). This potentially informs on the efficacy of using tDCS techniques within patient groups that have non-typical anatomy such as in adult (Datta et al., 2011; Dmochowski et al., 2013; Minjoli et al., 2017) and perinatal stroke (Gillick et al., 2014). It has previously been demonstrated in small samples that stroke-induced lesion damage can have marked effects on tDCS current flow patterns (Datta et al., 2011; Dmochowski et al., 2013; Gillick et al., 2014; Minjoli et al., 2017). Here we extend these findings with the largest pediatric sample to date. Our results demonstrate that peak EF strength appears to be higher in children with AIS compared to controls for conventional tDCS montages using active anodes over lesioned tissue (montages 1 and 3). While peak strengths were still within recommended safety margins (Bikson et al., 2016), especially when using moderate tDCS amplitudes such as 1mA, patient-specific hotspots may exist for some participants and suggests that tDCS-induced peak electric fields may be higher for children with perinatal AIS. By contrast, the HD-tDCS montage using an active anode over lesioned cortex (montage 4) showed lower peak EF strength for AIS compared to both PVI and TDC groups. This finding is likely because the more focal field, typical of HD montages, is being induced proximate to particularly conductive CSF-filled areas in the AIS group. These findings reinforce the need to consider patient-specific differences in dosing brought about by differing electric field patterns in perilesional and more remote areas.

Field focality was also found to be different across montages and patient groups. While it is relatively well established that HD-tDCS montages provide more focal stimulation than conventional tDCS montages in participants with intact cortex (Datta et al., 2009; Dmochowski et al., 2011), we can provide additional insight into perinatal stroke-related differences. We have demonstrated lower volumes of stimulated GM for the AIS group for montages using active anodes over lesioned cortex (montages 1, 3, and 4). This is likely due to lower volumes of brain tissue and higher volumes of CSF in close proximity to the anode as reflected by associations with lesion volumes found in this group. While the scalp to GM surface distance was higher for the AIS group, likely reflecting the presence of larger lesions, we did not find an association between this distance and field focality. Rather, measurements of lesion volume were more highly associated with EF focality. By contrast, field focality values were not different between groups for the montage using an active cathode over non-lesioned cortex (montage 2), likely due to the presence of intact cortex and relatively thin CSF spaces under the active electrodes, consistent with previous studies quantifying effects of CSF on field characteristics (Opitz et al., 2011, 2015).

Within target M1 regions of interest, Mean EF was lower for the AIS group compared to controls for montages using active anodes over lesioned cortex (montages 1 and 4) but were comparable or higher for montages using active cathodes over non-lesioned cortex (montages 2 and 5). Similar findings have been found for modeling of transcranial magnetic stimulation (TMS) induced electric fields in perinatal stroke suggesting that EF strength in lesioned tissue is lower than non-lesioned and that the variability of EF strength is larger in the lesioned hemisphere over non-lesioned (Mantell et al., 2021). CSF is particularly conductive and has a significant effect on resulting tDCS field characteristics (Datta et al., 2009; Bikson et al., 2012b; Opitz et al., 2015) especially in large CSF-filled lesioned areas after stroke (Datta et al., 2011; Gillick et al., 2014; Minjoli et al., 2017) perhaps allowing current to dissipate in CSF rather than reaching brain tissue in some cases. In other cases, conductive CSF may concentrate current in perilesional areas, again illustrating participant-specific idiosyncrasies in current propagation (Datta et al., 2011). Each of the above observations appear to support previous justifications for choosing to target the non-lesioned hemisphere in perinatal stroke trials (Hilderley et al., 2019).

Electric field angle varied by montage, consistent with past literature (Evans et al., 2022), and also showed montage-specific differences between participant groups. Cathodal montages (both conventional and high-definition) with target cortices located in the non-lesioned hemisphere showed lower EF angle values for TDC compared to the PVI group. The bihemispheric montage targeting the lesioned hemisphere additionally showed lower angle values for TDC compared to both PS groups. Since the orientation of EF in cortex has been shown to be associated with both cortical excitability (reflected in transcranial magnetic stimulation-induced motor evoked potential (MEP) amplitudes) (Rawji et al., 2018) and subsequent behavioral changes after tDCS interventions (Hannah et al., 2019), these participant- and montage-specific differences likely have implications for clinical applications of tDCS in patient populations. These findings also have implications for personalization of tDCS montages in participants with idiosyncratic lesions. Our results suggest that using simulations to individually characterize both EF strength and EF angle is important. Additionally, we had initially hypothesized that EF angle variability in the PS groups would have been higher than the TDC group due to heterogeneity of lesion architecture, but this was not the case, the groups showing equal variances. In addition, the reason for the absence of group differences in the anodal montages targeting lesioned tissue is also unclear and somewhat surprising given the large amount of anatomical variability in the lesioned hemisphere. Perhaps, since our anodal montage used a supraorbital return electrode, effects of lesions were smaller given the primarily anterior-posterior current propagation. Lesions may have had more impact on EF angles in montages using more laterally placed montages with primarily lateral-medial current propagation (such as our bihemispheric montage), as has been previously demonstrated in individuals without stroke (Evans et al., 2022). Performing precise, participant-specific EF simulations combined with applying tDCS montage optimization algorithms may help to further elucidate individual variability and perhaps improve intervention response in patients with CP-induced perinatal stroke.

### Anatomical characteristics

As hypothesized, individual anatomical characteristics were highly associated with field characteristics in a montage-specific way. Generally, montages using active anodes over lesioned cortex (montages 1, 3, and 4) appeared most sensitive to differences in underlying anatomy. While significant differences in model results have been noted when skull thickness and composition is taken into account (Opitz et al., 2015), we also note that variations in GM and WM tissue volume, as well as lesion volume were associated with field focality and EF strength within the target M1 regions. Montages using active cathodes over non-lesioned tissue (montages 2 and 5) seemed most stable and field characteristics were not associated with variations in underlying tissue and skull characteristics.

### Periventricular venous infarction

While the majority of differences in field characteristics were seen in the AIS group, differences were also demonstrated between the PVI group and controls. Children with PVI have largely intact cortices given that PVI-induced damage is typically constrained to periventricular areas, though significant alterations in ventricle morphometry and overlying cortical volumes can occur (Li et al., 2012). EF hotspots, as measured *via* peak EF, are seen in periventricular areas particularly for montages targeting lesioned hemispheres (illustrated in Figure 3B), compared to the control group. We also show higher mean EF strength in the GM of the target M1 region for montage 2, using a cathode in the non-lesioned hemisphere. Given that current spreads widely across the brain using conventional tDCS, alterations in ventricular morphometry in the PVI group may contribute to group differences. While these differences are not as striking as in the AIS group, it does suggest that CSF-filled areas such as dilated ventricles may still alter current flow in children with perinatal stroke even if those CSF-filled areas are not directly under active electrodes leading to periventricular “hotspots.” Patient-centered tDCS-induced EF simulations can thus also inform intervention customization in children with PVI-induced CP, not just AIS.

### Other perinatal stroke-specific considerations

In light of previous results (Ciechanski et al., 2018), we used diffusion-weighted imaging to calculate participant-specific anisotropic conductivities. This is especially important when applying simulations to a pediatric population given the relatively steep developmental trajectory of WM microstructure changes between 6 and 19 years of age and into early adulthood (Lebel and Beaulieu, 2011). Using diffusion-weighted anisotropic conductivity values has been shown to modulate simulations (Shahid et al., 2014; Ciechanski et al., 2018) though has a cost in terms of computational complexity. In children with perinatal stroke, previous studies have shown additional differences in myelination as measured by T1-weighted WM contrast in both the lesioned and non-lesioned hemispheres remote from the lesion (Yu et al., 2018). The presence of diaschisis, the degradation of brain remote from but anatomically connected to a lesioned area, has also been identified (Kirton et al., 2016b) which could potentially alter tDCS current propagation. Areas of cortical atrophy and/or thickened skull may also occur as a compensatory response after unilateral cortical injury significantly changing EF strength. For example, Dyke-Davidoff-Masson syndrome (Atalar et al., 2007) is characterized by hypertrophy of the skull ipsilateral to cortical atrophy and has been observed after perinatal stroke. Given these widespread, idiosyncratic anatomical differences after focal injury, it seems prudent to use the highest resolution and highest quality scans possible including personalized diffusion-weighted tensors defining anisotropic WM conductivity values even at the expense of simulation complexity and processing time.

### Implications for tDCS intervention efficacy

There remains much uncertainty as to the specific underlying mechanisms mediating tDCS-induced effects on behavior. Combinations of other external factors such as compliance, intensity of rehabilitation (number of repetitions), as well as subject factors (genetic determinants of plasticity, fatigue, motivation) and the multitude of possible tDCS configurations will also affect efficacy of tDCS interventions. Theoretically, it seems logical to focus highest peak EF strength on “target” cortical regions such as M1 to enhance motor performance, however, in reality it may be more beneficial to provide a more widespread increase (or decrease) in cortical excitability to entire motor-related networks rather than focal nodes of such networks. Future studies investigating underlying tDCS mechanisms will likely strive to characterize such montage-specific differences in efficacy. In addition, challenges with accuracy of mathematical FEM models, interpretability of resulting simulations, and technical considerations such as the quality of the scans and accuracy of resulting segmentations also need to be taken into account (Bikson et al., 2012b). Add to this the large heterogeneity in stroke locations and sizes in this population, and the challenge is clear. Even with this in mind tDCS EF simulations, if performed with sufficient attention to detail, may provide useful information as to which montage may be most beneficial for each patient in an individualized manner. Advanced algorithms have been developed to customize electrode placements based on individualized anatomy to optimize dosing (Dmochowski et al., 2013; Gillick et al., 2014). As these algorithms are further developed it seems personalized tDCS montage design in interventional trials may become more commonplace. Post-intervention simulations may also be informative to identify current propagation and tDCS dosage in responders versus non-responders to inform future clinical trials (Datta et al., 2011; Dmochowski et al., 2013). Additional technical refinements in simulation techniques will likely facilitate accuracy in predicting and maximizing efficacy.

### Limitations and future directions

There are limitations to this study that should be considered. Automated tissue segmentations were occasionally incorrect, resulting from head motion contamination or extensive lesion damage. These challenges could also have been exacerbated given that existing segmentation tools are typically optimized for young, healthy adults. As a result, 24 participants (∼20%) were excluded thereby reducing statistical power and generalizability to the entire population. These exclusions were mostly younger participants with larger lesions, thus the results presented here are likely a conservative estimate of the effects of age, tissue, and lesion volumes on tDCS EF simulations. While no attempt was made to manually edit the segmentations in the current study (i.e., participants were simply excluded), moving forward, it may be feasible to do so with future developments in modeling software and by using T1- and T2-weighted images as a reference. Stroke lesions are typically dark on T1 and bright on T2 imaging and thus would provide a sound basis for manually recoding segmentations. Concurrently viewing an age-matched control when recoding segmentations would provide an additional reference and use of an adaptive brush tool, accounting for surrounding tissue image contrast would reduce subjectivity. In the current study, we necessarily excluded participants with segmentation misclassifications as we did not want to introduce additional subjectivity *via* manual editing of segmentations. We also did not include compact and spongy bone as separate tissue classes despite differing conductivities and effects on EF strength in resulting simulations (Opitz et al., 2015). Meninges (Weise et al., 2022) and other idiosyncratic anatomical factors (Mosayebi-Samani et al., 2021) likely also affect EF simulations but were not explicitly investigated here. FEM calculations are an estimate and typically there is no ground truth available for comparison, however, studies have validated modeled versus true EF strength using intracranial cortical and depth electrodes in epilepsy patients (Huang et al., 2017; Puonti et al., 2020). We also used previously established tissue conductivity values optimized for use with adults (and not expressly optimized for children). We used the direct mapping approach for diffusion tensors in estimating anisotropic conductivity values, something that is also not optimized for children but validated in adults. Reverse phase-encoded diffusion sequences were not acquired and were therefore not utilized during the tensor calculation.

## Conclusion

Performing simulations of tDCS-induced EF strength using individualized MRI brain anatomy is feasible but challenging in children with large perinatal stroke lesions. We demonstrate that tDCS montages utilizing active anodes over lesioned cortex show differences in EF strength for children with AIS compared to controls. Montages using active anodes over lesioned cortex appear to be more sensitive to variations in underlying anatomy than montages using active cathodes over non-lesioned cortex. Individualized tDCS EF simulations, taking into account idiosyncrasies in brain architecture after stroke, may therefore be valuable for customizing therapeutic tDCS applications to achieve personalized rehabilitation interventions in future clinical trials.

## Data availability statement

The raw data supporting the conclusions of this article will be made available by the authors, without undue reservation.

## Ethics statement

The studies involving human participants were reviewed and approved by University of Calgary Research Ethics Board. Written informed consent to participate in this study was provided by the participants’ legal guardian/next of kin.

## Author contributions

HC, AG, and PC collected and analyzed data. HC drafted initial manuscript. All designed and conceptualized study, interpreted results, and edited and approved final submitted manuscript.

## Footnotes

2
www.hiccupkids.ca

3
https://simnibs.github.io/simnibs/build/html/index.html

4
http://www.neuro.uni-jena.de/cat

5
https://www.fil.ion.ucl.ac.uk/spm/software/spm12
