# Supplementary material for: Electric field simulations of transcranial direct current stimulation in children with perinatal stroke
Source: Front Hum Neurosci. 2023 Feb 2;17:1075741. doi: 10.3389/fnhum.2023.1075741 (PMC9932338; doi:10.3389/fnhum.2023.1075741)
Supplement: Supplementary file 1 [file Data_Sheet_1.docx]

**Supplementary Materials**

**Table S1.** Electrode placements using the EEG 10/20 system for five montages targeting primary motor cortices.

| **Montage**  **Laterality of stroke** | **Anode** | **Cathode** | **Cathode** | **Cathode** | **Cathode** |
| --- | --- | --- | --- | --- | --- |
| Montage 1 – Anodal tDCS of M1_Les_ | | | | | |
| Left stroke  Right stroke | C3  C4 | Fp2  Fp1 |  |  |  |
| Montage 2 – Cathodal tDCS of M1_NonLes_ | | | | | |
| Left stroke  Right stroke | Fp1  Fp2 | C4  C3 |  |  |  |
| Montage 3 – Bihemispheric tDCS of M1_Les_ and M1_NonLes_ | | | | | |
| Left stroke  Right stroke | C3  C4 | C4  C3 |  |  |  |
| Montage 4 – Anodal HD-tDCS of M1_Les_ | | | | | |
| Left stroke  Right stroke | C3  C4 | CP5  CP6 | FC5  FC6 | FC1  FC2 | CP1  CP2 |
| Montage 5 – Cathodal HD-tDCS of M1_NonLes_ | | | | | |
| Left stroke  Right stroke | C4  C3 | CP6  CP5 | FC6  FC5 | FC2  FC1 | CP2  CP1 |

Table note: Right hemisphere stroke configurations were used for control participants. HD - high-definition.

**White Matter Analyses**

**Group differences in electric field strength and focality for white matter**

For WM (Table S2), montage 1 showed the highest peak EF strength for PVI over the TDC group (t_(80)_=2.7, p=0.008) with montages 2 and 3 showing no group differences. Montage 4 showed higher peak EF values for both the TDC (t_(80)_=3.2, p=0.002) and PVI groups (t_(80)_=4.0, p<0.001) over the AIS group. Montage 5 showed higher peak EF for the AIS group over the TDC group (t_(80)_=2.3, p=0.022).

In montages 1 and 3, the volume of WM tissue above 75% of the 99.9^th^ percentile for EF was larger for the TDC group compared to both the AIS (montage 1: t_(80)_=4.7, p<0.001; montage 3: t_(80)_=2.4, p=0.019) and PVI groups (montage 1: t_(80)_=4.7, p<0.001; montage 3: t_(80)_=2.9, p=0.005). Montage 5 showed higher volumes for the TDC over the AIS group (t_(80)_=2.4, p=0.017). For current density, higher WM volume values were seen for montage 3 in the TDC group compared to both AIS (t_(80)_=3.4, p=0.001) and PVI groups (t_(80)_=3.0, p=0.004). For montages 4 and 5, the TDC group showed larger volume values over that of AIS (montage 4: t_(80)_=3.0, p=0.004; montage 5: t_(80)_=3.2, p=0.002).

**Table S2.** White matter peak electric field, current density and field focality for five tDCS montages by participant group

| **Values** - mean (SD) [min-max] | **AIS (N=21)** | **PVI (N=30)** | **TDC (N=32)** |
| --- | --- | --- | --- |
| Peak electric field (V/m)  Montage 1 - Anodal tDCS of M1_Les_  Montage 2 - Cathodal tDCS of M1_NonLes_  Montage 3 - Bihemispheric tDCS  Montage 4 - Anodal HD-tDCS of M1_Les_  Montage 5 - Cathodal HD-tDCS of M1_NonLes_ | 0.42 (0.08) [0.28-0.62]  0.41 (0.07) [0.29-0.62]  0.45 (0.09) [0.30-0.70]  0.14 (0.08) [0.07-0.35]  0.24 (0.12) [0.14-0.69] | 0.44 (0.10) [0.32-0.66]  0.40 (0.06) [0.29-0.52]  0.46 (0.10) [0.34-0.67]  0.21 (0.08) [0.12-0.45]  0.22 (0.09) [0.10-0.43] | 0.40 (0.05) [0.30-0.51]  0.40 (0.05) [0.29-0.53]  0.42 (0.06) [0.30-0.53]  0.19 (0.06) [0.10-0.34]  0.18 (0.06) [0.08-0.32] |
| Electric field focality (cm^3^)  Montage 1 - Anodal tDCS of M1_Les_  Montage 2 - Cathodal tDCS of M1_NonLes_  Montage 3 - Bihemispheric tDCS  Montage 4 - Anodal HD-tDCS of M1_Les_  Montage 5 - Cathodal HD-tDCS of M1_NonLes_ | 8.1 (3.4) [1.7-14.6]  15.0 (5.1) [2.9-23.3]  11.4 (4.6) [2.7-25.3]  4.0 (2.2) [1.1-9.8]  3.9 (1.5) [1.1-6.8] | 8.8 (5.7) [1.7-24.5]  14.1 (4.5) [6.1-26.2]  10.3 (4.8) [2.0-19.3]  4.0 (1.8) [1.3-10.3]  4.1 (2.0) [0.9-9.2] | 13.3 (3.8) [7.0-21.0]  13.0 (3.8) [6.6-19.2]  14.0 (4.0) [6.6-21.0]  4.6 (1.6) [1.8-8.9]  5.1 (1.9) [1.9-9.0] |

Table note: tDCS - transcranial direct current stimulation, AIS – Arterial ischemic stroke, PVI – Periventricular venous infarction, TDC – Typically developing controls, GM - grey matter, WM - white matter, HD - high-definition tDCS. Electric field strength (EF) is reported in V/m. Peak EF values reported correspond to the 99.9^th^ percentile value. Field focality is the tissue volume (in cm^3^) that had EF values ≥ 75% of the 99.9^th^ percentile.

**Group differences in M1 regions of interest for white matter**

EF values for WM in the M1 ROI differed across participant groups. For montages 1 and 3, AIS showed the lowest values compared to TDC (montage 1: t_(80)_=3.6, p<0.001; montage 3: t_(80)_=3.5, p=0.001) and PVI (montage 1: t_(80)_=3.8, p<0.001; montage 3: t_(80)_=3.2, p=0.002). For montage 2, AIS showed higher EF values compared to TDC (t_(80)_=3.2, p=0.002). For montage 2, AIS showed lower EF values than PVI (t_(80)_=2.9, p=0.004).
